# Data reconstruction can improve abundance index estimation: An example using Taiwanese longline data for Pacific bluefin tuna

**DOI:** 10.1371/journal.pone.0185784

**Published:** 2017-10-02

**Authors:** Shui-Kai Chang, Hung-I Liu, Hiromu Fukuda, Mark N. Maunder

**Affiliations:** 1 Institute of Marine Affairs, National Sun Yat-sen University, Kaohsiung, Taiwan; 2 Overseas Fisheries Development Council of the Republic of China, Taipei, Taiwan; 3 National Research Institute of Far Seas Fisheries, Japan Fisheries Research and Education Agency, Shizuoka, Japan; 4 Inter-American Tropical Tuna Commission, La Jolla, California, United States of America; Universita degli Studi di Bari Aldo Moro, ITALY

## Abstract

Catch-per-unit-effort (CPUE) is often the main piece of information used in fisheries stock assessment; however, the catch and effort data that are traditionally compiled from commercial logbooks can be incomplete or unreliable due to many reasons. Pacific bluefin tuna (PBF) is a seasonal target species in the Taiwanese longline fishery. Since 2010, detailed catch information for each PBF has been made available through a catch documentation scheme. However, previously, only market landing data with a low coverage of logbooks were available. Therefore, several nontraditional procedures were performed to reconstruct catch and effort data from many alternative data sources not directly obtained from fishers for 2001–2015: (1) Estimating the catch number from the landing weight for 2001–2003, for which the catch number information was incomplete, based on Monte Carlo simulation; (2) deriving fishing days for 2007–2009 from voyage data recorder data, based on a newly developed algorithm; and (3) deriving fishing days for 2001–2006 from vessel trip information, based on linear relationships between fishing and at-sea days. Subsequently, generalized linear mixed models were developed with the delta-lognormal assumption for standardizing the CPUE calculated from the reconstructed data, and three-stage model evaluation was performed using (1) Akaike and Bayesian information criteria to determine the most favorable variable composition of standardization models, (2) overall *R*^*2*^ via cross-validation to compare fitting performance between area-separated and area-combined standardizations, and (3) system-based testing to explore the consistency of the standardized CPUEs with auxiliary data in the PBF stock assessment model. The last stage of evaluation revealed high consistency among the data, thus demonstrating improvements in data reconstruction for estimating the abundance index, and consequently the stock assessment.

## Introduction

The catch rate or catch per unit effort (CPUE) expresses the number of fish caught with a certain amount of fishing effort; it is frequently used as an index of relative fish abundance [1]. Catch and effort data for CPUE calculation are traditionally compiled from commercial logbooks, but data from this source can be incomplete, unreliable, or error prone [1–3] because of commercial confidentiality or unwillingness to report. Landing data can be used as an alternative to catch information with additional assumptions, such as no discarding [4–6]. However, effort data calculated from logbooks are commonly suspect for fisheries research purposes because of the nature or unreliability of the data [1,3]; and the situation might be worse when dealing with small fisheries, for which logbook reporting, historically not a mandatory practice, tends to provide limited coverage. Therefore, alternative approaches are required to address this concern for understanding resource trends. One approach is to derive high-quality effort data from high-tech information systems, such as a vessel monitoring system (VMS), coastal surveillance radar system, or voyage data recorder (VDR) system [5–10].

Pacific bluefin tuna (PBF, *Thunnus orientalis*) are highly migratory in the temperate areas of the North Pacific Ocean (NPO), with occasional appearances in the Southern Pacific Ocean near Australia and New Zealand [11,12]. They mature at 4 years of age [13,14] and spawn separately around the Ryukyu Islands and in the Sea of Japan during late April to early August [15–17]. Hatched larvae migrate northward to the nursery areas off the southern or western coasts of Japan in summer [18–21]. PBF aged 1–2 years exhibit either seasonal east—west movement around the southern Japanese coast or a combination of staying in the East China Sea and moving toward the Kuroshio—Oyashio transition region [21–23]. Many fish perform trans-Pacific migration to the eastern NPO starting from the age of 1–2 years [22,24], exhibit repeated northwest–southeast movement between the waters off Mexico and those off central California, migrate back to the western NPO at the age of 3–4 years, and later gather in the two spawning grounds for reproduction [21,25]. After spawning, adults in the western NPO typically migrate northward to feeding grounds, with an unknown proportion moving toward the south or east [26].

**Fig 1:**
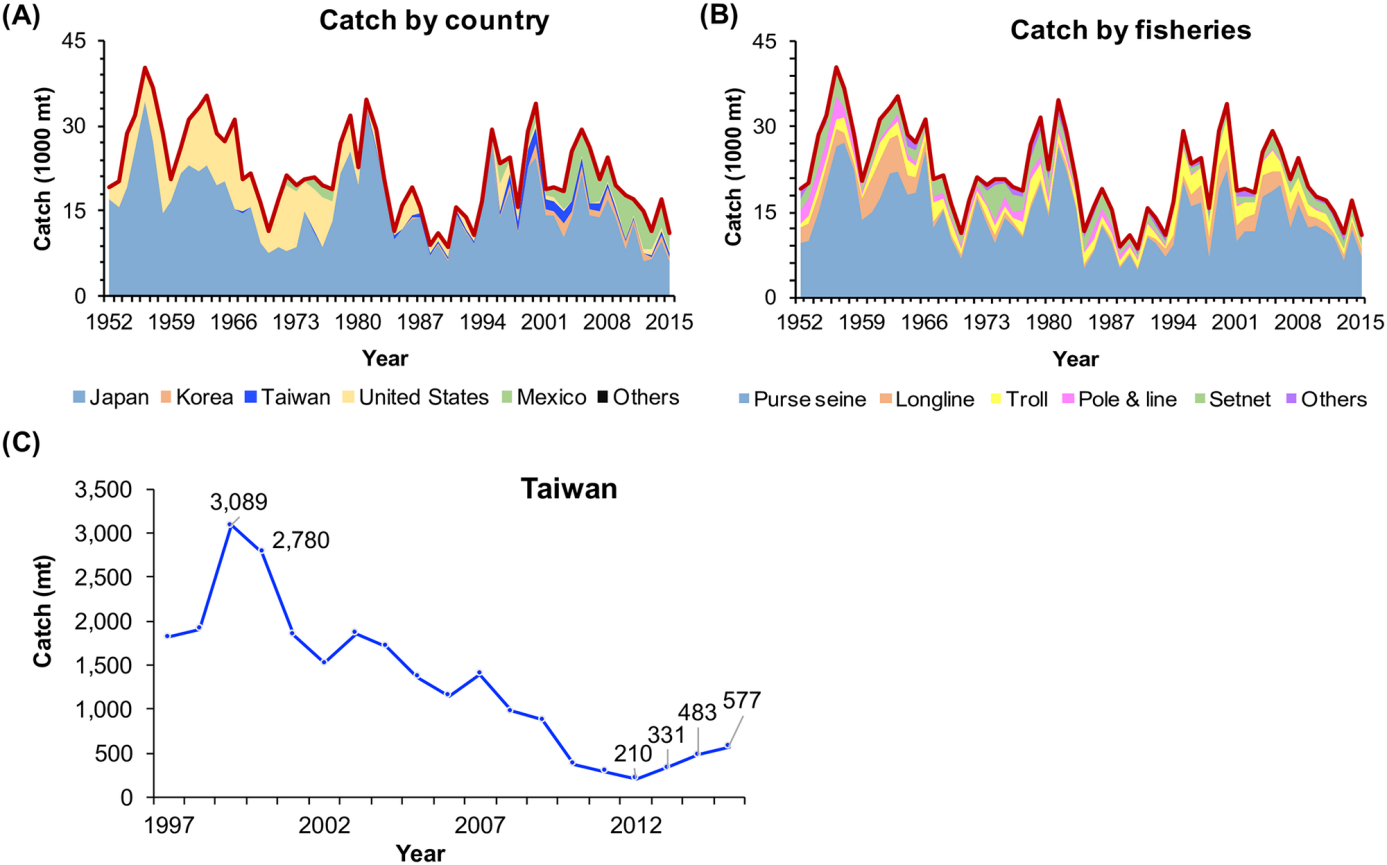
PBF catch series. Panels (A) and (B) indicate PBF catches for 1952–2015 by the country and fisheries, respectively. Panel (C) shows the catch by the Taiwanese longline fishery during 1997–2015; data for 2015 are preliminary. The data are provided in S1 File.

PBF is assumed to be a unit stock in the Pacific Ocean and has been extensively exploited at all life stages throughout its distribution range by many countries because of its high commercial value [13] (Fig 1, S1 File). Adult spawning fish are caught by the longline fisheries of Taiwan and Japan and the Japanese purse seine fishery in the western NPO. Juvenile fish are caught by the Japanese troll, purse seine, and setnet fisheries; Korean purse seine fishery; Mexican purse seine fishery; and USA sport fishery. The highest annual catch of PBF was recorded in 1956. Thereafter, this annual catch has shown three major peak-to-lowest cycles during periods when global management regulations were not implemented: 1963–1970, 1981–1990, and 2000–2013 (Fig 1). The peak catches in all cycles were similar (nearly 35,000 mt). The lowest catches were nearly 9,000–11,000 mt, and the time from the lowest level to the peak was around 10 years during the period.

Regular stock assessments of PBF have been conducted by the International Scientific Committee for Tuna and Tuna-Like Species in the North Pacific Ocean (hereafter, ISC). The latest three assessments in 2012, 2014, and 2016 concluded that the stock is overfished and overfishing is occurring relative to the commonly used biological reference points for tuna stocks. However, the 2016 assessment suggested that the stock has slightly recovered from the recent lowest level [13,27]. Currently, only the longline fishery provides reliable information from which a relative abundance index for the spawning stock can be derived. Taiwan and Japan are the only countries to use longline gear for targeting the spawning stock. Although the Taiwanese catch is relatively small (approximately 2% since 2010), data from the Taiwanese longline fishery are important for assessment because the fishery has the only fleet operating during a short season in the southern boundary area of the spawning aggregation of the largest sized fish (>200-cm fork length [FL]) [28]. The data from this fleet could provide information on the relative trend of the largest fish as well as the upper bound of the population size [29]. However, in the 2014 assessment, the Taiwanese data were suspected as a major cause of poor model performance—inadequate fits and conflicts among the Taiwanese and Japanese longline CPUE series and their associated size compositions [27]. Maunder et al. [30] pointed out the inconsistency of the information between Taiwanese longline CPUE trend and its size composition data. For example, the CPUE increased several years after the strong cohort appeared in the size composition data of the fishery. These concerns may have emerged because of incomplete data for deriving a reliable CPUE series.

PBF is an important seasonal target species for the Taiwanese offshore longline fishery. The PBF catch peaked in 1999 (3,089 mt), continuously declined to reach the lowest level in 2012 (213 mt), and rebounded to slightly more than twice that level by 2014 (Fig 1). The catch in Taiwan’s tuna fisheries is relatively small; therefore, logbook submission was not required until 2010, when specific management regulations were implemented on PBF fishery. The regulations require all PBF vessels to join the catch documentation scheme (CDS) to report every PBF catch to a nearby fishery radio station, providing information on the catch date, location, and weight estimate and provide a CDS document for the port inspector to verify and measure the fish length and weight. Moreover, since 2007, all vessels intending to apply for fuel subsidies from the government have been required to install a VDR [10], and since 2010, all PBF vessels larger than 20 gross registered tonnage (GRT) have been required to install a functional VMS. However, before 2010, only landing data since 2001 with 100% coverage in weight and logbook data with only a small fraction of the trips (<5%) were available. The catch and effort data required for developing a PBF CPUE time series were incomplete for Taiwanese fishery.

This study is to address the lack of data by reconstructing catch and effort data using data sources not directly obtained from fishers and to obtain a reliable abundance index by standardizing the CPUE calculated from the entire series of the reconstructed data, for the Taiwanese PBF fishery. CPUE standardization aims to explain and exclude the variation in the CPUE that does not result from changes in abundance by identifying explanatory variables that reduce the unexplained variation in the response variable [31]. Many types of standardization models are available, and Hinton and Maunder [32] proposed three categories of methods to evaluate model performance. The first two are based on the ability to predict the catch or CPUE by assuming that the models most accurately predicting the mentioned factors are the most efficient predictors of relative abundance. The third category is based on the consistency of the estimates, with auxiliary information on the year effect that represents the annual relative levels of abundance.

First, this study presents nontraditional procedures to reconstruct the catch and effort data for 2001–2005 by (1) estimating the PBF catch number from landing weight for 2001–2003 when information on the catch number was incomplete, based on Monte Carlo simulation; (2) deriving fishing days for 2007–2009 from the VDR, based on a newly developed approach, and (3) deriving fishing days for 2001–2006 from vessel trip information, based on the linear relationship between fishing and at-sea days within a trip. Furthermore, generalized linear mixed models (GLMMs) were developed with a delta-lognormal assumption for standardizing the CPUE series. A three-stage model evaluation was performed using methods introduced by Hinton and Maunder [32]: (1) the Akaike information criterion (AIC) and Bayesian information criterion (BIC) [31], (2) cross-validation and bootstrap [33] for estimating the overall coefficient of determination (*R*^*2*^), and (3) system-based testing involving the advanced PBF stock assessment model adopted by the ISC [13]. These tests not only determined the most efficient CPUE standardization model but also revealed that the data reconstruction and CPUE standardization model improved the estimation of the abundance index of PBF in Taiwan and addressed the concerns of inconsistency indicated by the ISC [27,30].

## Materials and methods

### Data source

**Fig 2:**
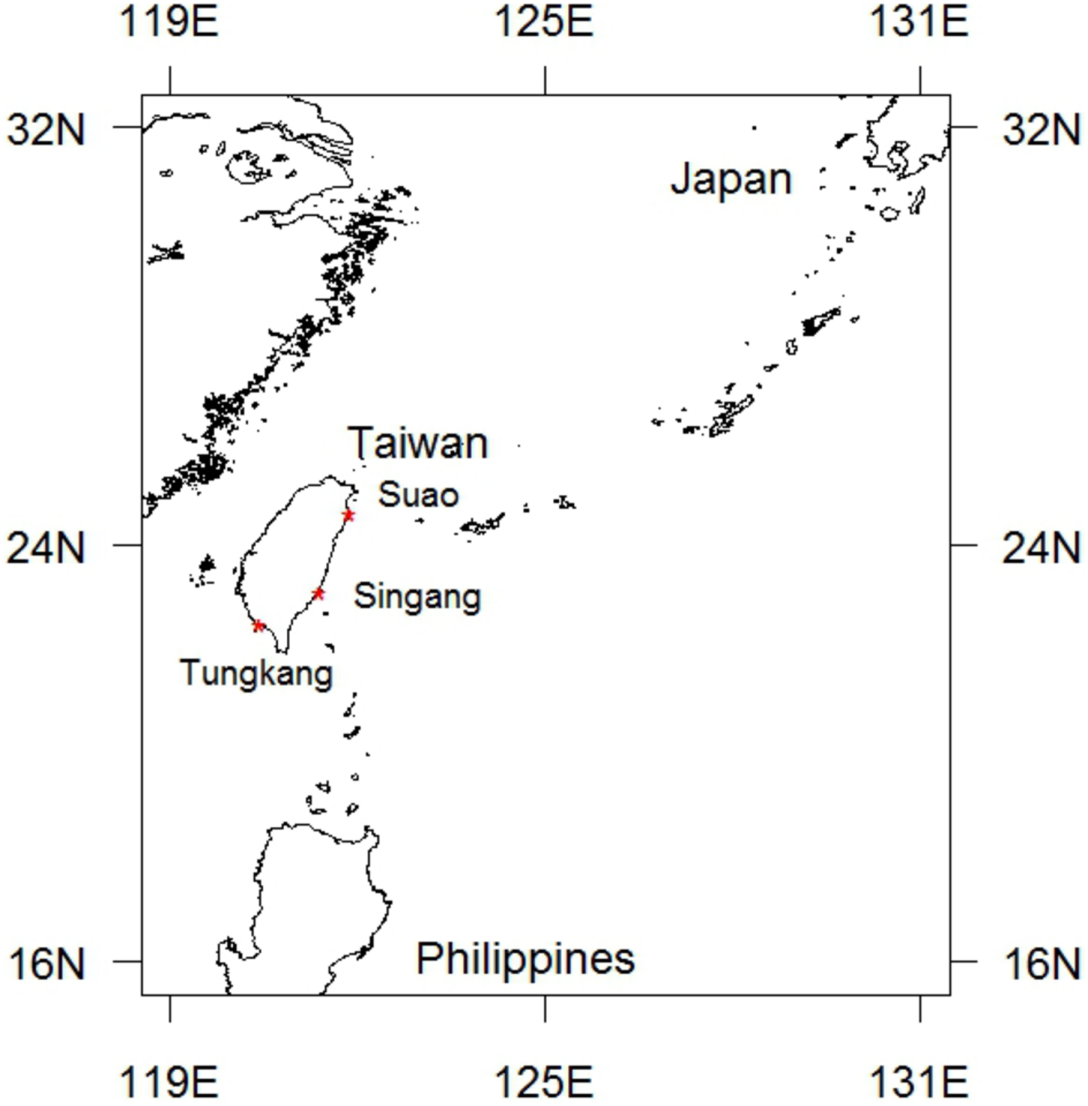
Major PBF landing ports in Taiwan: Suao, Singang, and Tungkang.

In this study, six types of data were collected to estimate the PBF CPUE for the Taiwanese longline fishery (Table 1). All the data were provided by the Fisheries Agency, Taiwan. The first two types of data were used for estimating PBF catches. The first type was a complete set of CDS data with detailed information on PBF catches during 2010–2015. The second was a series of market landing data from 2001 to 2015, with landing information by the vessel, landing date and port. PBF catches by the longline fishery were all offloaded in three major fishing ports (markets) of Taiwan, namely (in descending order of the landing amount) Tungkang, Suao, and Singang (Fig 2). The CDS data having spatiotemporal information on each PBF catch was mainly used for verification and to separate the catch based on the catch location. The fish number was considered the catch in CPUE calculation. However, the number of fish offloaded in 2001–2003 was incomplete and needed to be estimated from the landing weight, which was complete for the entire data series.

**Table 1:** Data types used in this study for estimating PBF CPUE. Available data periods and remarks on data contents and limitations are provided.

Data type	Period	Remarks
Catch documentation scheme (CDS) data	2010–2015	Includes complete information on the catch date, position, length and weight of each PBF, and supplemental information on vessels and auctions.
Market landing data	2001–2015	Includes the landing weight and number of PBF offloaded by the vessel, landing date, and port (fish market). Information on the number of fish was incomplete for 2001–2003.
Logbook data	2001–2015	Includes information on the operation date and location, fishing effort, and catch by species. The coverage was very low until 2010.
Vessel trip data	2001–2015	Contains date and time information on fishing vessels leaving and entering the major fishing ports in Taiwan, by the vessel and port. The data were collected by the Coast Guard Administration of Taiwan for security purposes.
Voyage data recorder (VDR)data	2007–2015	Contains records of the vessel position, speed, and direction in 3-min increments. The data were originally obtained from offshore and coastal vessels applying for fuel subsidy from the government.
Vessel monitoring system (VMS) data	2007–2015	Contains records of the vessel position, speed, and direction in 1–6-h increments. The number of vessels with the VMS was low before 2010.

The third type of data is logbook data. However, logbook coverage was very low (<5%) until 2010 when the new regulations were implemented. Effort information from logbook data was used only for verification purposes, as explained in a later section.

The remaining three types of data were used for estimating fishing effort. Considering the difficulties in estimating the number of hooks through incomplete data, this study used fishing days as a measure of fishing effort. PBF vessels include four size categories in terms of GRT: CT1 for 5–10 GRT, CT2 for 10–20 GRT, CT3 for 20–50 GRT, and CT4 for 50–100 GRT [6,10]. According to the logbook data, the number of hooks deployed per day for registered PBF longliners differed among the vessel size categories, but were similar within categories (cv = 26%–30%). The number of hooks can be calculated from the mean number of hooks deployed per day by the vessel size, if necessary, but will introduce additional variation.

The fourth type of data was vessel trip data containing records of vessels leaving and entering fishing ports in Taiwan, by the vessel and port, during 2001–2015. The data were collected by the Coast Guard Administration (CGA) of Taiwan for security purposes [6].

The fifth and sixth types of data were VDR and VMS data for 2007–2015, containing vessel codes, positions, speeds, and directions. The position data of the VDR and VMS were obtained every 3 min and 1–6 h, respectively. VDR data were originally used to estimate the distance that the offshore and coastal vessels traveled at sea so that the vessels could apply for fuel subsidies from the government [10]; thus, the coverage was high. VMS data were used for compliance purposes according to PBF fishery regulations. The number of PBF vessels with VMS was low before 2010, and the data were mainly used to supplement the VDR data to obtain geolocation data (still referred as the VDR data) for a more complete estimation of fishing days.

### Methods

Four tasks were performed in this study to reconstruct catch and effort data and to obtain a reliable CPUE series: (1) estimate fish numbers for 2001–2003, (2) derive fishing days from VDR data for 2007–2009, (3) derive fishing days from vessel trip data for 2001–2006, and (4) develop models to standardize the CPUE series and perform three-stage model evaluation for 2001–2015. Task (1) aimed to obtain complete information on the fish number series, and tasks (2) and (3) aimed to complete the effort data series. All calculations except that for stock assessment were performed using SAS version 9.4 [34].

#### (1) Fish number estimation for 2001–2003

**Fig 3:**
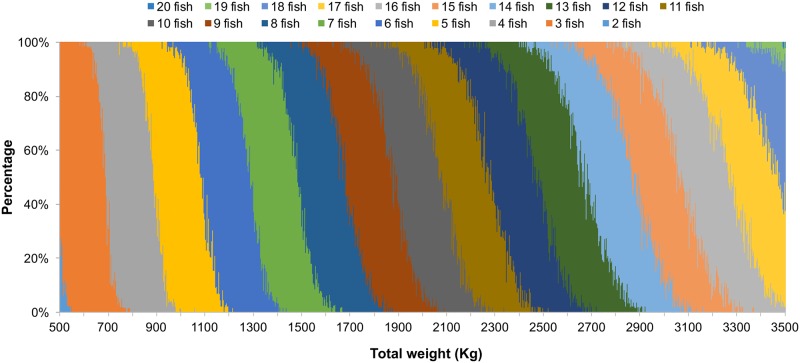
Simulated weight distribution by fish number. Each color band represents the weight distribution of a certain number of fish. For example, the left gray band shows the weight distribution of four fish, with a total weight of 650–950 kg.

Only 20%–36% of landing records contained information on the fish numbers during 2001–2003. These data were mostly obtained from Suao and Singang, which have fish sizes different from Tungkang, the largest port; therefore, the data were considered nonrepresentative. The most recent 3-year data set (2004–2006) with complete fish number and weight information yielded the following findings. First, landing a single fish was common, possibly because of the high value and sparsity of the PBF catch, and the weight per fish (WPF) was 80–350 kg. Second, the annual WPF distribution showed normal or lognormal patterns. The Kolmogorov–Smirnov (KS) two-sample test ([35]) was subsequently used to determine the actual WPF distribution against normal and lognormal distributions to define the most suitable distribution. Thereafter, Monte Carlo simulation involving three steps was conducted to estimate the fish number for each year of 2001–2003.

i All landing records (on day and vessel basis) of 80–350 kg were selected, and a WPF distribution was constructed from the records by assuming each record stands for one fish.
ii Fish (1–50) were randomly selected from the WPF distribution and their weight were summed up to obtain the total weight of 1–50 fish. This practice was repeated 10,000 times, and a weight distribution by the fish number was constructed. Fig 3 presents the distribution between 2 and 20 fish for 2001.
iii The fish number was randomly selected from the weight distribution for the landing records without information on the fish number, according to the total landing weight by the day and vessel.

#### (2) Fishing day estimation from VDR data for 2007–2009

High-tech data, such as VMS data, have been used to determine fishing efforts in many fisheries [7,8]. A longline operation comprises two components: hook deployment (usually in the morning, at higher vessel speeds) and hook retrieval (typically in the afternoon until midnight, at lower vessel speeds). During operation, the distance covered by a vessel in 2 days or within a day would generally be small. Based on these observations, Chang and Yuan [7] tested four approaches, including a combination of two approaches, and recommended two of them as criteria for classifying fishing days from the VMS data of Taiwanese distant-water longliners: first, the optimal speed–time range approach, which is based on a vessel’s speed in the afternoon (the hook retrieval period), and second, the within-day-distance approach, which is based on a vessel’s moving distance within a day. The format of VDR data is similar to that of VMS data. The CDS and logbook data for 2010–2015 can provide information on whether the PBF fishing vessels were fishing on a specific day during the fishing season. Therefore, this study tested the two recommended approaches by using the VDR data of PBF vessels having both logbooks and CDS information.

Almost all PBF are caught by offshore longliners, which are more mobile and may have a different operation pattern than distant-water longliners; therefore, this study tested a newly designed approach. Based on consultations with onboard observers, offshore longliners have a clear pattern of direction change after hook deployment in preparation for hook retrieval. If the vessel was not fishing and was moving directly toward a location, the direction change in a day may be small or nearly 0 (i.e., a straight line). Therefore, the third approach tested was based on changes in the vessel direction.

In this study, several combinations of criteria were tested using the three approaches, and the optimal criterion was finally selected based on performance measures described in the following sections.

i Vessel speed approach: This approach was renamed from the “optimal speed–time range approach” described by Chang and Yuan [7] because a set of pretests showed no apparent speed differences between hook deployment and retrieval for the PBF longline fishery. This study tested the performance of the following criteria: a day with at least an instance of vessel speed at x knots was defined as a fishing day, where x = 1–7 knot(s)/knot.
ii Within-day distance approach: The study tested the criteria that a day can be defined as a fishing day if the within-day-distance was less than x km, where x = 70–190 km/10 km.
iii Direction change approach: The study tested the criteria that defines a day as a fishing day if the angle of the direction change was within x degrees; x = 5°–180° per 5°. To clearly determine the general change in direction, the 3-min position data must be compiled on a larger scale. Therefore, the study simultaneously determined the effect of time duration by selecting only the position data of every x h after recording the earliest data of the day for examining the direction change; x = 1–6.

The performance of the criteria was evaluated based on the ability to maximize the agreement between the predicted fishing and nonfishing day distribution from VDR data and the observed distribution from logbooks and CDS data by measuring the sum of sensitivity and specificity (SSS; the larger the better) and absolute difference in sensitivity and specificity (DSS; the smaller the better) [7,36–39]. Elements of the confusion matrix were denoted as true positive (TP), false negative (FN), false positive (FP), and true negative (TN). The sensitivity of the criterion [or the true positive rate, TPR = TP/(TP + FN)] was measured as the ability to accurately predict fishing days. Moreover, the specificity [or the true negative rate, TNR = TN/(TN + FP)] was measured as the ability to accurately predict nonfishing days [36,37]. The criterion that maximized the SSS and minimized the DSS, when the SSS was similar to others, was considered optimal [38,39].

These tests were performed using data recorded since 2010. Before all tests were conducted, the per 3-min VDR data were processed to per 1-h data. The optimal criterion with the most desirable performance was subsequently applied to the VDR data for 2007–2009 to derive fishing days for each PBF vessel.

#### (3) Fishing day estimation from vessel trip data for 2001–2006

The vessel trip data from the CGA can be used to calculate the at-sea days of each trip for each PBF vessel. A data exploration analysis of the data for years after 2010 (Table 1) suggested linear relationships between at-sea days (independent variable) and fishing days (dependent variable), by the vessel size and fishing port. The vessel trip data were first screened for accuracy and then used to establish the relationships from the data for 2007–2015. The linear relationships were constructed by the vessel size (CT1–CT4) and fishing port (three ports) and applied to the trip data for 2001–2006 to estimate fishing days.

#### (4) CPUE standardization and model evaluation for 2001–2015

Through the aforementioned data reconstruction procedures, a CPUE series for 2001–2015 was obtained. The CPUE must be standardized to eliminate the confounders affecting the representativeness of this CPUE as an appropriate abundance index [31]. A commonly applied model for CPUE standardization is the generalized linear model (GLM), which can account for changes in fishing practice in a linear pattern [31,40]. This study applied the GLMM, an extension of GLM, by considering some parameters in the linear predictor as random variables. The response variable in the model was the natural logarithm of the CPUE. The addition of more explanatory variables will typically increase the fraction of variability explained; however, it will also increase the variation in the abundance index [31]. No additional reliable information is available; therefore, this study simply considered three explanatory variables that might influence the catchability: year (2001–2015), month (May–July), and vessel size (CT1–CT4). PBF migrate to the adjacent waters off Taiwan during late April to July for spawning; therefore, only data obtained during May to July from the registered PBF vessels were used. The number of hooks deployed per day differed with the vessel size. Moreover, the catchability was also assumed to vary with the vessel size; therefore, the size category was included as an explanatory variable.

**Fig 4:**
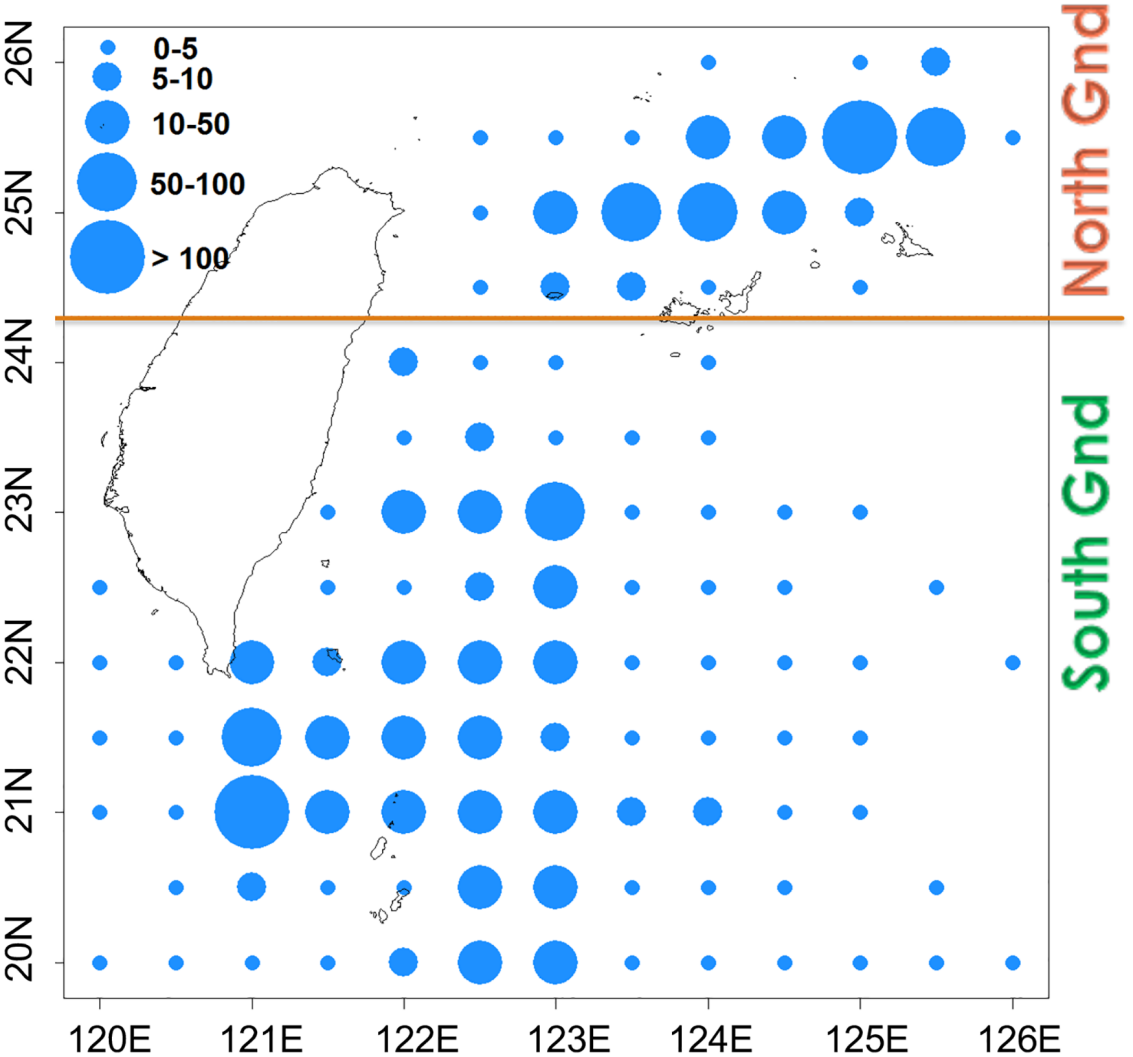
Average PBF catch distribution off Taiwan during 2010–2015 by Taiwanese PBF longliners. The line splits the fishing grounds into southern and northern areas at 24.3°N.

**Fig 5:**
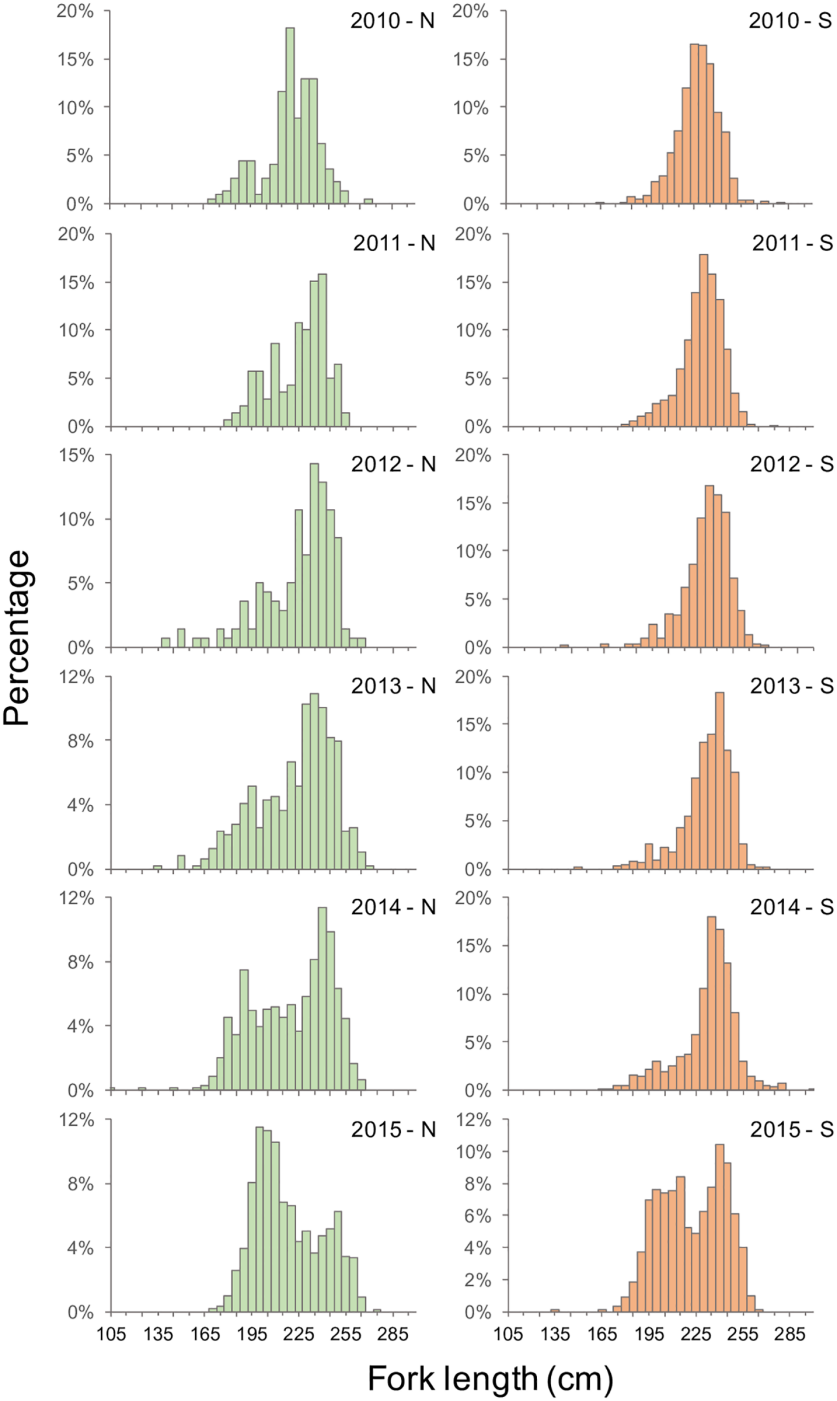
Length frequencies of PBF caught by the Taiwanese longline fishery. The length frequencies are presented as the FL in cm (bottom limit) in the northern fishing ground (left panel, green) and southern fishing ground (right panel, orange) during 2010–2015. Please refer to Fig 4 for the definition of fishing grounds. The data are provided in S2 File.

Taiwanese longliners operate in two major PBF fishing grounds, which are split at 24.3°N (Fig 4) [28]. Except for 2015, the PBF catch in the northern area has shown a wider size range (170–260 cm FL) than that in the southern area has (190–260 cm FL) and has been approximately 25 kg smaller on average (Fig 5, S2 File). Before 2008, when the PBF catch was still high (Fig 1), the southern area was exploited almost solely by vessels from Singang and Tungkang, whereas the northern area was exploited by vessels leaving Suao. Therefore, the catch was separated based on the vessel’s departure port recorded in the vessel trip data for 2001–2006 when no fishing location information was available. For data from 2007 onward, VDR and CDS information was used to separate the catch by area. With fishing ground information, this study performed three standardizations: a CPUE standardization for the entire fishing ground with the area (northern or southern) as a categorical factor (area-combined standardization) and two CPUE standardizations separated by area (area-separated standardizations).

Before the analyses were conducted, data were aggregated in advance on a trip basis, as followed for the Japanese PBF CPUE standardization [41], because no daily fishing information was available for data recorded before 2007.

PBF are infrequent in the catch; therefore, the CPUE data was composed of a high proportion of zero observations, which may violate the model assumptions and jeopardize the integrity of the inferences if not properly modeled [31]. This study applied a delta approach [42] to separately model the proportion of zero observations and observations with positive catches. The proportion of zero observations was modeled using a binomial distribution (zero-proportion model, ZPM), and the positive catch rate was modeled using a lognormal distribution (positive-catch model, PCM). Details of this approach have been previously reported [31,40,43]. The standardized index is calculated as the product of the year effects (least square means [LSmeans]) from each of the two model-estimated components [44–46]. A weighted factor proportional to the number of observations in the input data to account for the unbalanced characteristics of the data was used in the lognormal estimates of LSmeans, and bias correction was additionally applied to the estimates by using the algorithm reported by Lo et al. [42].

A small number of explanatory variables were considered in the study because of the limited information available. Therefore, a simple forward (increasing variables) then backward (decreasing variables) method was used for determining the variables to be included in the model. All three explanatory variables (year, month, and vessel size) were initially included in both models and were retained in the final models by using the backward method. Furthermore, first-order interactions of the explanatory variables were considered for both models and were determined using the forward method. The interactions of year and other categorical variables (month and vessel size) were considered random variables [31,44]. The analyses and model formulations were conducted using the GLMMIX and MIXED procedures in SAS.

Three methods corresponding to the three categories methods reported by Hinton and Maunder [32] were used to evaluate the CPUE models. In this study, these methods included three stages of model evaluations. The first stage was to select the most favorable variable combination (‘the final model’) for each of the three standardizations by using the AIC and BIC. The second stage involved defining the most efficient model design between the area-separated and area-combined standardizations based on the overall *R*^*2*^ value estimated through cross-validation and bootstrap procedures. The last stage involved investigating which of the three standardized CPUE series had the most favorable consistency with other data associated with PBF resources (e.g., size and other fisheries data), and thus could be considered as the abundance index of Taiwanese fishery, by applying the CPUE data to the PBF stock assessment model.

First, the AIC and BIC [31] obtained from the aforementioned statistical procedures were used to select the final model (with the smallest AIC and BIC values) from all model runs. The selection was conducted separately by one area-combined and two area-separated standardizations.

Second, standardization using the delta approach involves two steps, each yielding a set of AIC and BIC values; model selection can be applied independently for each step. However, comparing the AIC and BIC of the area-separated standardization with the AIC and BIC of the area-combined standardization is complicated because of difficulties in defining the variance parameters of the likelihood function and convergence concerns associated with area interactions. Therefore, to compare the performance of area-separated and area-combined CPUE models, the overall *R*^*2*^ value was estimated through cross-validation and bootstrap procedures [33,47]. *R*^*2*^ determines the correlation between the actual and predicted values and can be used for model selection in linear models [48]. However, a model may produce misleadingly high *R*^*2*^ because of overfitting. Cross-validation and bootstrap procedures were used to avoid the illusion of increased *R*^*2*^ caused by overfitting. The procedure involves four steps:

i The original data were randomly split into two data subsets through uniform distribution *U*(0,1) by considering the explanatory variables of year, month, area, and vessel size.
ii The first subset was considered the model-building set and was used to estimate the parameters (coefficients) of the three final sets of CPUE standardization models for the southern and northern fishing grounds and the combined fishing ground.
iii The second subset was considered the validation set and was used to calculate *R*^*2*^ for model evaluation. In this study, first, the “theoretical” (predicted) data points were calculated from the final models obtained in step (ii): A value of 0 or 1 for the first component of the delta approach (ZPM) was decided by comparing a random value from the standard uniform distribution [*U*(0,1)] with the predicted probability of zero from the ZPM obtained in step (ii). The value was then multiplied with the predicted CPUE obtained from the second component of the delta approach (PCM) obtained in step (ii). Thus, pairs of observed and predicted CPUE for all data in the validation set could be obtained and separated for each of the three sets of CPUE standardization models. The *R*^*2*^ value was then calculated from the pairs of observed and predicted CPUE for the area-separated model (combining pairs of data from the CPUE models for the southern and northern fishing grounds) and the area-combined model.
iv Steps i–iii were repeated 200 times to obtain the mean and 95% confidence intervals of *R*^*2*^ for the area-separated and area-combined models.

The third stage involved system-based testing by applying the final standardized CPUE series from the first stage to the latest PBF stock assessment model. The model was established by the ISC PBF Working Group in 2016 [13], which was an annual time-step length-based, age-structured, forward simulation population model developed using Stock Synthesis version 3.24f [49], which is freely provided at http://nft.nefsc.noaa.gov/Stock_Synthesis_3.htm. S3 File provides detailed information on the model structures, fishery data and assumptions, and biological assumptions required for performing the PBF stock assessment. The model and model input were further described in an assessment report [13]. The CPUEs of the Taiwanese longline fishery estimated in this study and those of Japanese longline fishery estimated in a previous study [41] were considered the “observed” relative abundance indices of large adult fish (age: >8 years). A series of scenarios (sensitivity runs) on those CPUEs were tested to determine how the CPUE series performed as abundance indices in the stock assessment model. We compared model fits to the longline CPUEs and size composition data to investigate the consistency (compatibility) of the estimated CPUEs with regard to the other data sources in the assessment model. The model fit to the data was evaluated with the root mean square error (RMSE) between the aforementioned observed indices and indices predicted by the assessment model, as well as the calculated negative log likelihood for the size composition data component. The following scenarios were tested.

1. Taiwanese longline southern CPUE and Japanese longline CPUE were fitted in the model.
2. Taiwanese longline area-combined CPUE and Japanese longline CPUE were fitted.
3. Taiwanese longline southern and northern CPUE and Japanese longline CPUE were fitted.
4. Only the Japanese longline CPUE was fitted.
5. Only the Taiwanese longline southern CPUE was fitted.

## Results and discussion

### Fish number estimation for 2001–2003

**Fig 6:**
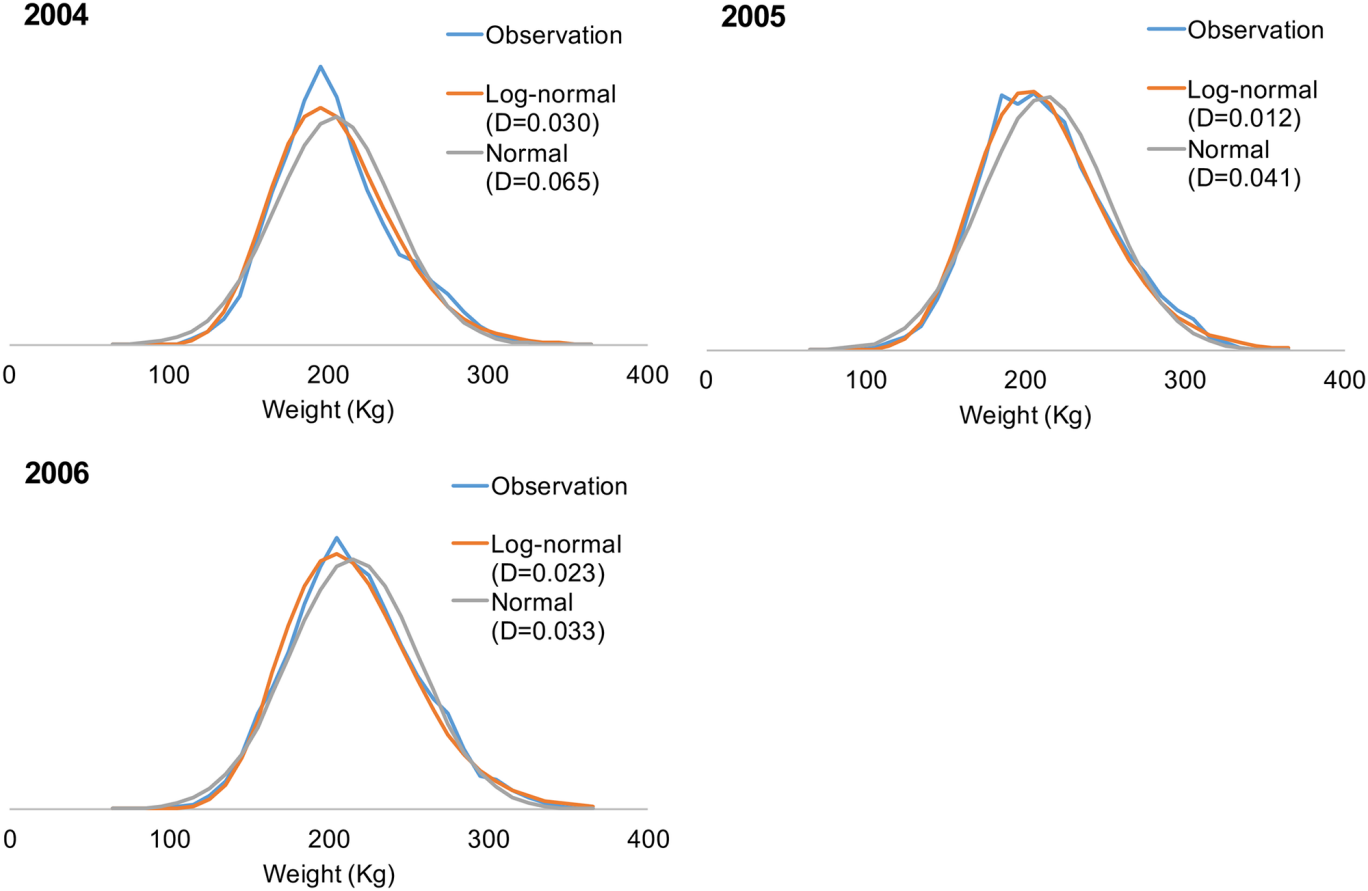
Actual and estimated PBF weight distributions. The distributions were calculated from the actual fish numbers in market landing data and from estimated fish numbers through Monte Carlo simulation for 2004–2006.

**Fig 7:**
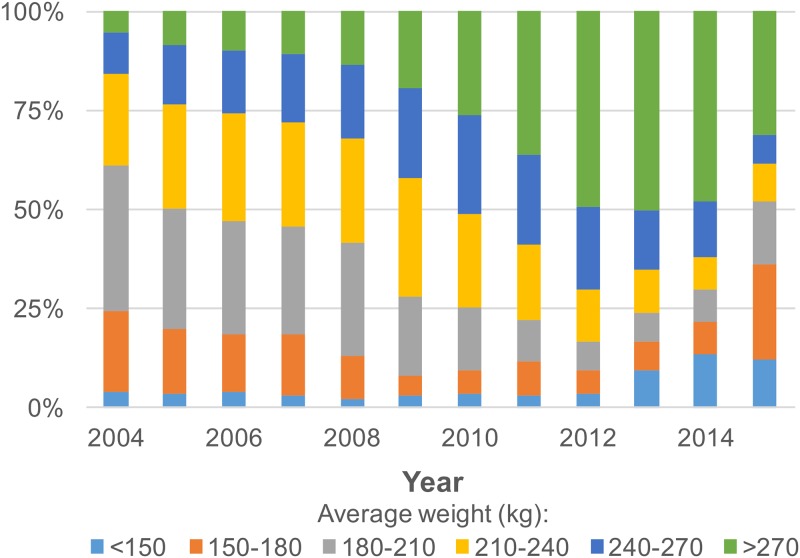
Percentages of average weight by the category of Taiwanese PBF catches from 2004 to 2015. The average weight was calculated from historical landing data with information on both catch number and weight. Data are provided in S4 File.

Monte Carlo simulation has numerous applications in fisheries [50]. The approach was applied in this study to estimate the number of fish in the catch according to the weight data for 2001–2003. A probability distribution is required to determine the fish size distribution in the simulation; both normal and lognormal distributions were previously assumed for fish size distributions [51]. The lognormal distribution has an elongated right-hand tail, accounting for the small number of disproportionately large individuals [52] typically observed in PBF catches. The KS two-sample tests on the WPF distribution of 2004–2006 data indicated no significant differences between the actual and assumed normal or lognormal distributions (p > 0.10; Fig 6), suggesting that both distribution assumptions were acceptable. However, based on the plots and the KS test D statistic (Fig 6), lognormal distribution with a smaller D statistic (0.012–0.030 for lognormal distribution compared with 0.033–0.065 for normal distribution) was more preferable. The D statistic was consistently smaller for the lognormal assumption than for the normal assumption in each year during 2004–2006, even there was a general declining trend in fish size in the period (Fig 7, S4 File). Therefore, lognormal distribution was adopted for the Monte Carlo simulation runs.

After the distribution assumption was determined, the simulation was tested on the 2004–2006 data to compare the actual fish number distribution with the simulated one, and the KS test revealed no statistical differences between them (D = 0.009–0.010, p = 0.932–0.993). The Monte Carlo simulation was subsequently applied to estimate the catch number for each year during 2001–2003.

### Fishing day estimation from VDR data for 2007–2009

**Fig 8:**
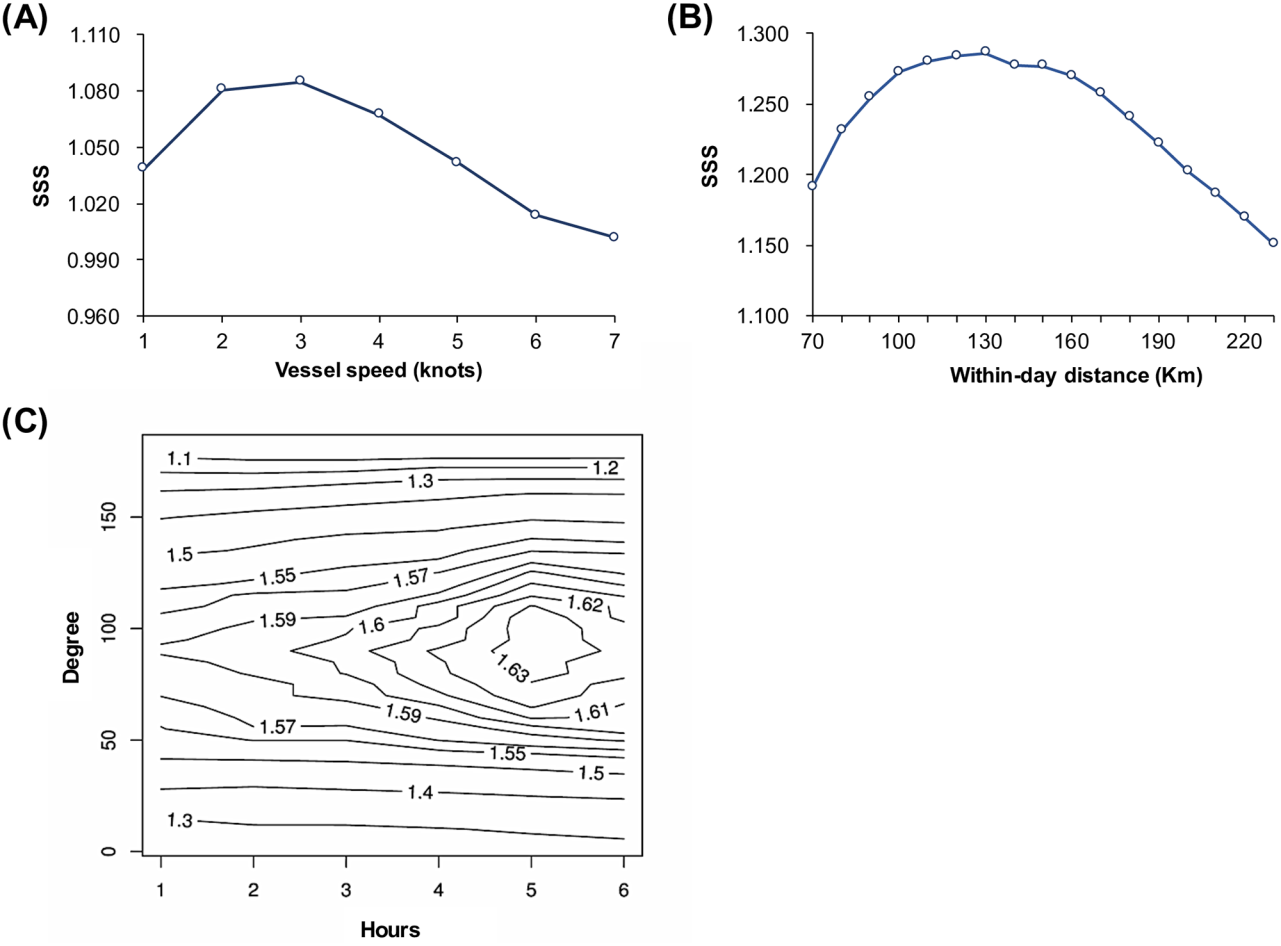
Distributions of the SSS. Panel (A) is the SSS obtained from the vessel speed approach and (B) is that from the within-day distance approach for developing the fishing day determination criteria. Panel (C) is the SSS contour plot at different data collection intervals (1–6 h) and direction change angles (5°–180°) for the direction change approach.

This task essentially defined the suitable fishing day classification criteria from the VDR data of the fishery. The application of the three fishing day classification approaches to the 2010–2015 VDR data revealed the performance statistics of the criteria (SSS), indicating the following maximum values: a speed of 3 knots for the within-day vessel speed approach (Fig 8A), a daily movement of 130 km for the within-day distance approach (Fig 8B), and a change in vessel direction of 90° in a day for data in 5-h increments for the direction change approach (Fig 8C). The statistics of the confusion matrix for the three optimal choices (Table 2) indicate that the direction change approach has the highest SSS and lowest DSS and therefore was recommended as the optimal criterion for determining fishing days from the VDR data for 2007–2009.

**Table 2:** Performance statistics of fishing day versus nonfishing day binary classification criteria, based on (A) vessel speed, (B) within-day distance, and (C) direction change approaches.

	TP	FN	FP	TN	TPR	TNR	SSS	DSS
***A*. *Vessel speed approach***								
3 knots	2731	96	1031	136	0.966	0.117	1.083	0.850
***B*. *Within-day distance approach***								
130 km	2482	343	690	474	0.879	0.407	1.286	0.471
***C*. *Direction change approach***								
90° per 5 h	2687	202	342	841	0.930	0.711	1.641	0.219

Note: True positive (TP), false negative (FN), false positive (FP), true negative (TN), true positive ratio (TPR), and true negative ratio (TNR) are shown, as well as their sensitivity and specificity (SS). The sum of the SS (SSS) and absolute difference in SS (DSS) are used as performance measures for the criteria.

**Fig 9:**
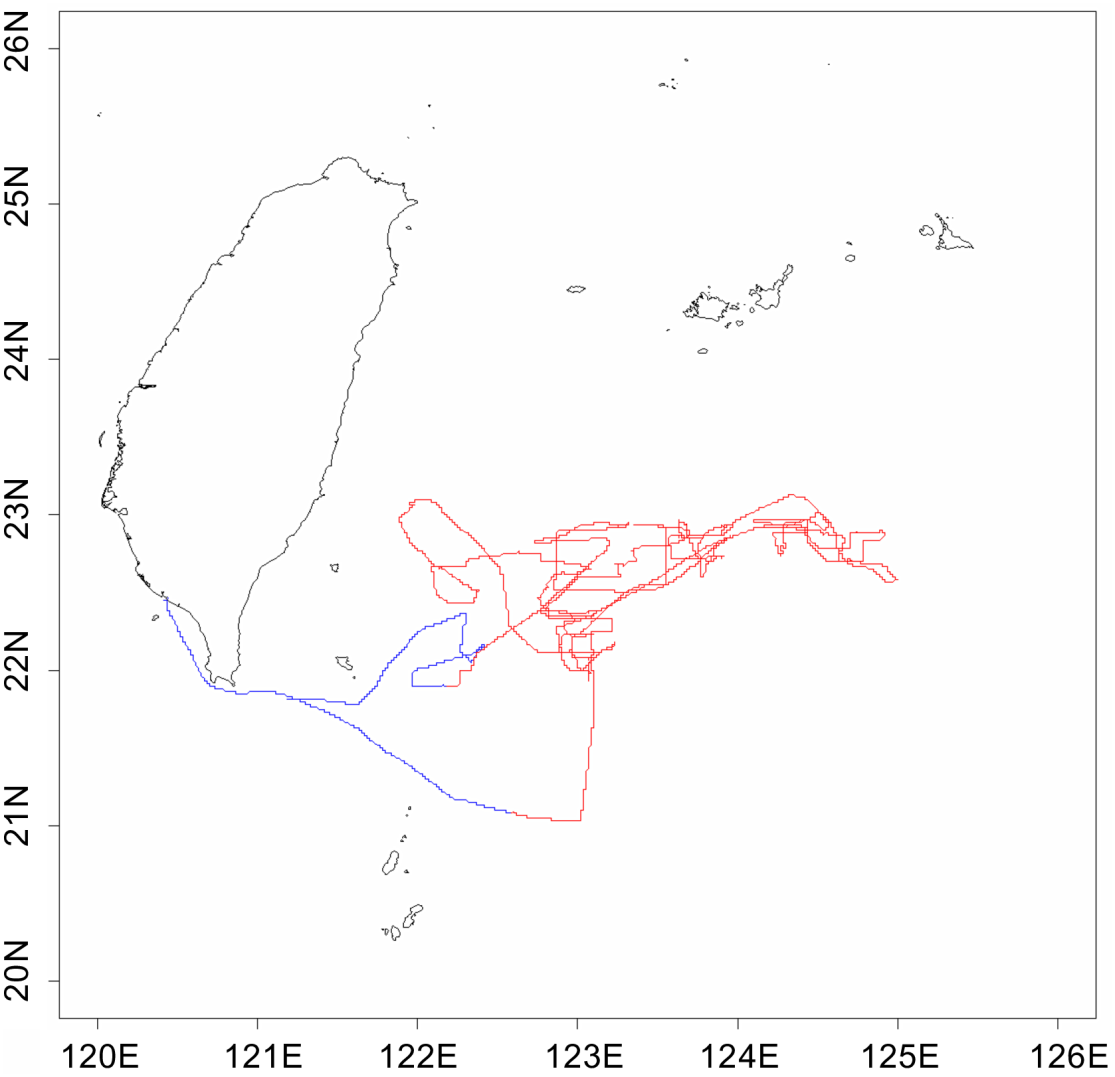
Navigation tracks of a PBF vessel. The red line indicates the fishing status and the blue line indicates the nonfishing status, as classified from the optimal direction change criterion.

**Fig 10:**
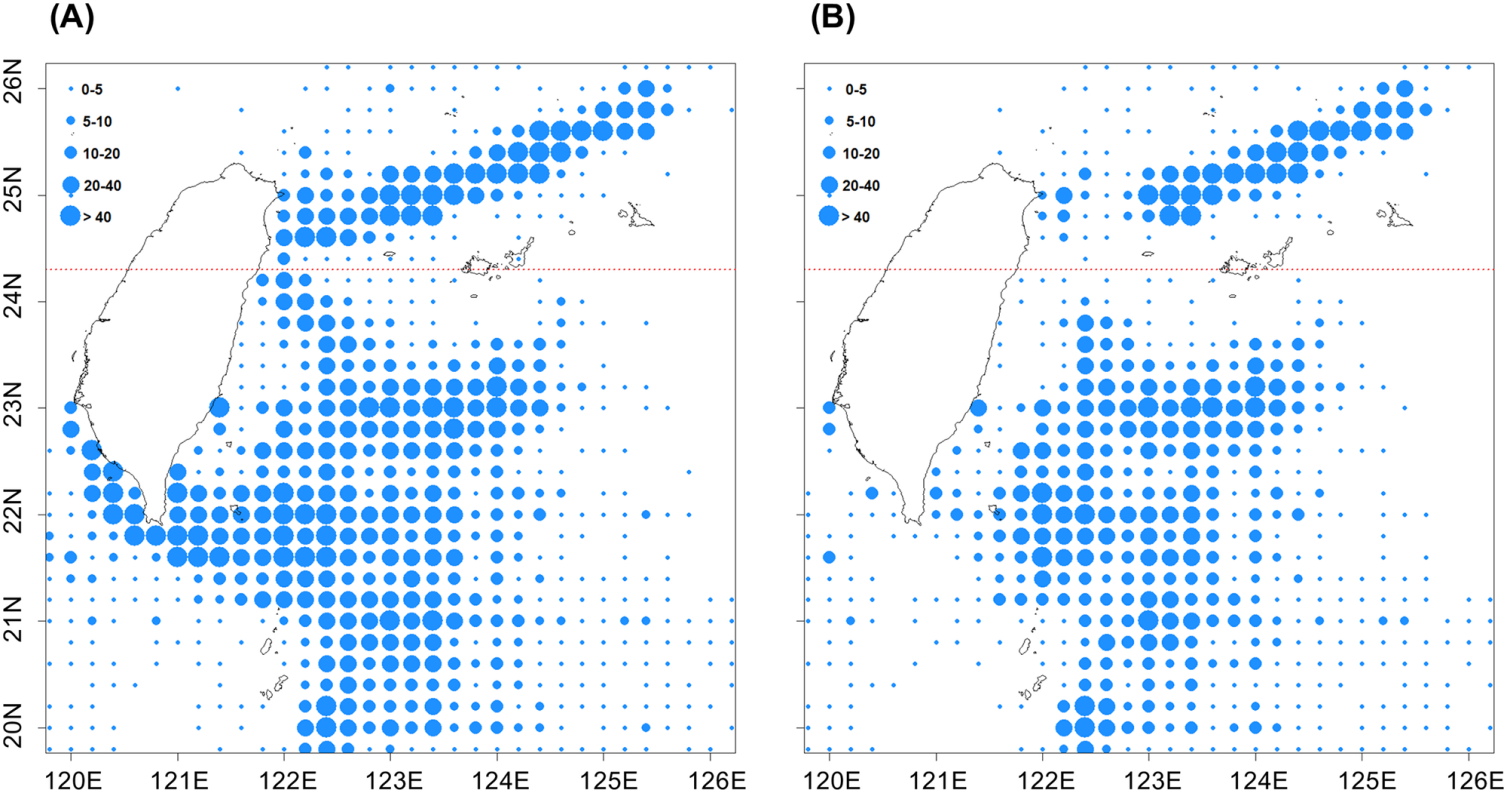
Distributions of at-sea days (left) and fishing days (right) of PBF vessels for 2007. The fishing days are classified according to the optimal direction change criterion.

An example plot of the tracks of nonfishing and fishing definitions based on the optimal criterion (Fig 9) revealed that the nonfishing period apparently occurred when the vessel was navigating to and returning from the fishing ground. The application of the optimal criterion (Fig 10) showed a comparison of at-sea and fishing day distributions for 2007 and suggested that the fishing day classification removed a substantial portion of at-sea days that were considered not fishing.

Studies exploiting existing independent geolocation data to develop fishing day classification criteria and then deriving fishing effort data from the criteria have become popular since the mid-2000s [8,53,54]. Those studies have mostly used satellite-based VMS data and have been conducted on trawl or dredge fisheries by using simple speed criteria; when the vessel speed is within a specific range, it is considered to be fishing [8,9]. Moreover, studies on the purse seine fishery were initiated in the 2010s through advanced approaches, including state-space models and artificial neural networks [55,56]. Chang and Yuan [7] were the first to study the approaches on distant-water longline fishery, passive fishing gear, and use a combination of fishing speed and period criteria (optimal speed–time range approach); when the vessel speed in a specific period is within a specific range, it is considered to be fishing. They also used vessels’ moving distance criteria (within- and between-days distance approaches); when a vessel’s moving distance within a day or between 2 consecutive days is within a specific range, it is considered to be fishing.

In addition to the VMS, Chang [6] used another source of data, the coastal surveillance radar system, which provides free information on the vessel position and speed, to determine the fishing effort of coastal fisheries. However, the system needs additional mechanisms to provide the vessel identity, and the data quality is easily degraded by unfavorable weather conditions. Chang [10] introduced another system, the VDR, which provides spatiotemporal information similar to that provided by the VMS and radar system, but has the major advantages of low installation cost, no transmission cost, and higher data resolution (at 3-min intervals; see [10] for detailed comparative discussions). The VDR, which was originally intended to evaluate fuel subsidies for Taiwanese offshore fisheries, was reported to be useful for estimating high-resolution fishing effort for trawl fisheries [10].

This study applied the approaches for developing fishing day criteria that were recommended by Chang and Yuan [7] and used the VDR data of the PBF longline fishery to estimate the fishing effort. They tested four approaches, and the optimal criterion was developed from the optimal speed–time range approach, which is at least one VMS report with a speed within 2–5 knots (optimal speed) detected during 14:00–23:00 h (namely, a time range). However, as previously mentioned, for the PBF offshore fishery, no apparent speed differences were observed between hook deployment and retrieval operations; therefore, the “time range” component is not applicable in this fishery. This probably resulted from the relatively smaller fishing area and shorter operation period of the offshore fishery; the average number of hooks per set was 700–1000 for CT2–CT4-sized longliners, compared with 3000–3600 hooks for distant-water longliners [7]. Therefore, the approach without the time range component was renamed the vessel speed approach in this study.

The direction change approach showed the optimal performance among the three approaches, with much higher SSS and lower DSS. A longline operation occurs in two parts: hook deployment and, after a short break for bait soaking, hook retrieval. After hook deployment, the vessel may start the hook retrieval operation from the position of the end of the deployment (starting retrieval from the latest deployed hooks) or return to the beginning position of the deployment (starting from the earliest deployed hooks). Irrespective of the type, the vessel must change the head direction substantially; this can be used for developing fishing day classification criteria. This approach was not previously tested on a distant-water longline fishery operating in a large area [7]. The direction change might not be notable for a distant-water fishery because the sea current may change the positions of both longline set and vessel. However, future studies must apply this approach to distant-water and longline fisheries targeting other tuna species.

### Fishing day estimation from vessel trip data for 2001–2006

Fishing days are typically calculated from logbooks. In many cases, at-sea days are used or proposed to represent fishing days when logbooks are insufficient [57,58]. However, this method is mainly suitable for when the fishing ground is near a port (coastal fisheries). At-sea days may include days for traveling, searching, and other vessel activities [59] and thus may not be a direct proxy for fishing effort. Fishing days can be estimated from high-tech GPS data (in this case, VDR data) with geolocation information for incomplete data situations. For the years without geolocation data, the relationship between at-sea and fishing days must be determined. When the relationships are being established, factors such as the vessel size (associated with moving power) and fishing port (associated with the distance from the fishing ground) must be considered.

An analysis of variance (ANOVA) was performed to investigate the relationship between the response variable of the fishing days estimated from the VDR data and the explanatory variables of the at-sea days calculated from the vessel trip data, by the vessel size and port, for 2007–2015 (Table 3, S5 File). The ANOVA indicated a highly significant relationship and significance effects of all explanatory variables. Therefore, simple linear relationships between the at-sea and fishing days were established by the vessel size and port, assuming that no at-sea days imply no fishing days (zero intercept in the regression model; Table 3). The 11 linear relationships (three vessel size categories for Suao and four for Singang and Tungkang) were statistically significant at the 1% level with *R*^*2*^ > 90%. The coefficients were subsequently applied to the vessel trip data for 2001–2006 to estimate the fishing days from at-sea days by the vessel size and port.

**Table 3:** Analyses of variance for testing the significance of the linear relationships between at-sea and fishing days. The relationships were established from data for 2007–2015 and were statistically significant at the 1% level.

**Analyses of variance**					
**Source**	**DF**	**Sum of squares**	**Mean square**	**F value**	**Pr > F**
Model	6	878261	146377	40395	<.0001
At-sea days	1	875811	875811	241691	<.0001
Port	2	2066	1033	285	<.0001
CT	3	384	128	35	<.0001
Error	9807	35537	4		
Total	9813	913798			
**Statistics for linear relationships**					
**Ports**	**Vessel size**	**Coefficient**	***R***^***2***^ ^†^		
Suao	CT2	0.828	0.967		
	CT3	0.824	0.975		
	CT4	0.797	0.962		
Singang	CT1	0.818	0.955		
	CT2	0.816	0.952		
	CT3	0.831	0.963		
	CT4	0.791	0.963		
Tungkang	CT1	0.750	0.974		
	CT2	0.810	0.970		
	CT3	0.752	0.968		
	CT4	0.693	0.934		

^†^ *R*^*2*^ was calculated using SAS version 9.4 under the condition of zero intercept, which may result in the overestimation of *R*^*2*^ [61]. *R*^*2*^ was 0.80–0.91, as calculated using Microsoft Excel.

Vessels leaving and entering Taiwanese ports must register for inspection by the CGA for security purposes because of the special antagonistic relationship between Taiwan and China [60]. The data are the only information that can be used for calculating at-sea days for 2001–2006, when VDRs had not yet been installed on coastal and offshore vessels. The clear linear relationships between at-sea and fishing days by the vessel size and port could be explained by the fact that different-sized vessels have different capacities for staying at sea and that the distance to the fishing grounds varies among fishing ports. For example, the coefficients were larger for Suao (0.80–0.83) than for Tungkang (0.70–0.81; Table 3), suggesting that, in general, vessels leaving Suao spent approximately 8 of 10 days fishing and 2 days traveling. Moreover, vessels leaving from Tungkang spent approximately 1 more day traveling, probably because the fishing ground was farther and broader (Fig 10).

### CPUE standardization and model evaluation for 2001–2015

#### (1) Standardization and first-stage model evaluation

**Fig 11:**
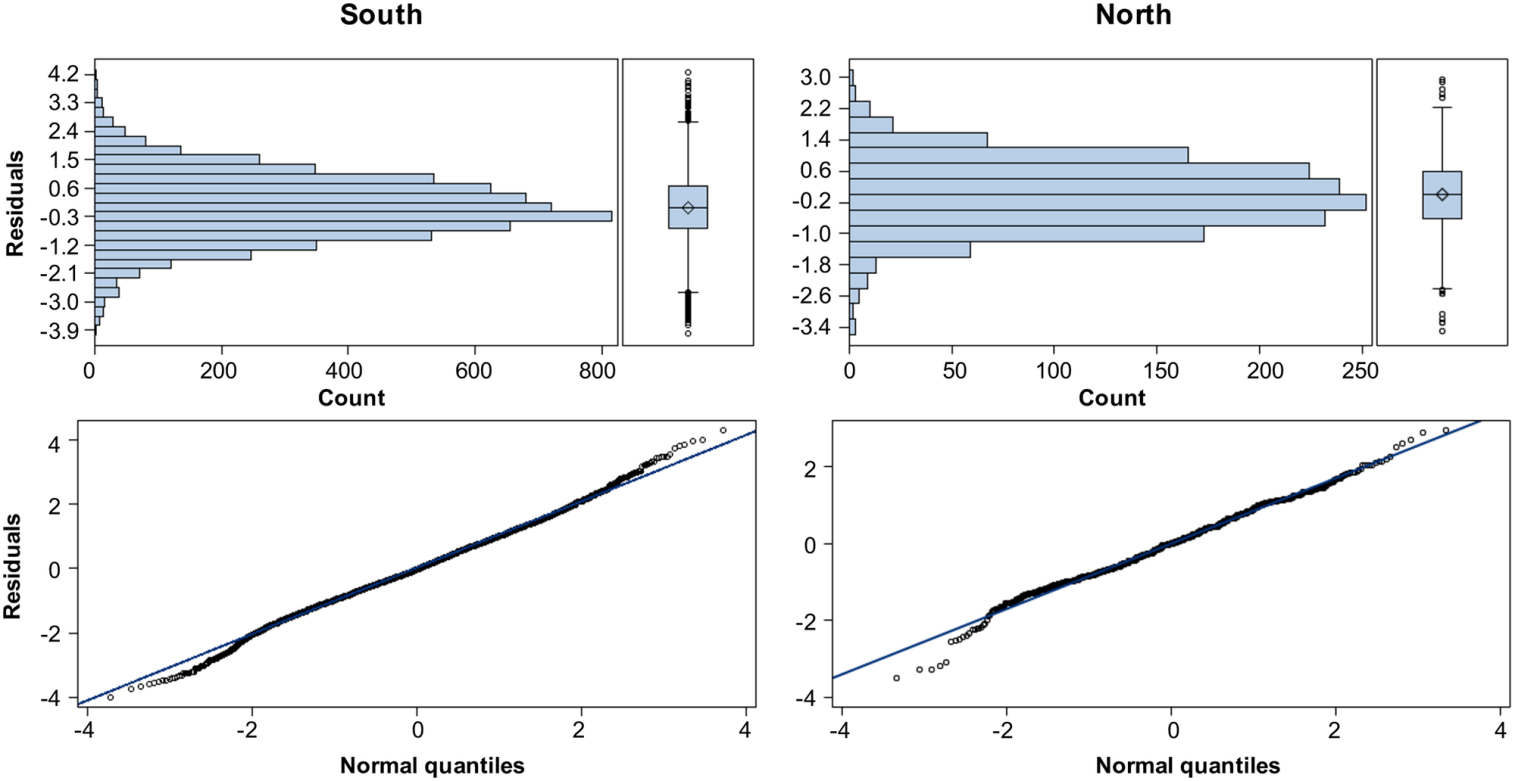
Diagnostic residual plots. The plots are for the GLM runs with a delta-lognormal assumption for the standardization of the PBF CPUE for the southern (left) and northern (right) fishing grounds. The KS two-sample tests indicate that both residual distributions do not significantly differ from the normal distribution assumption (p > 0.10).

**Fig 12:**
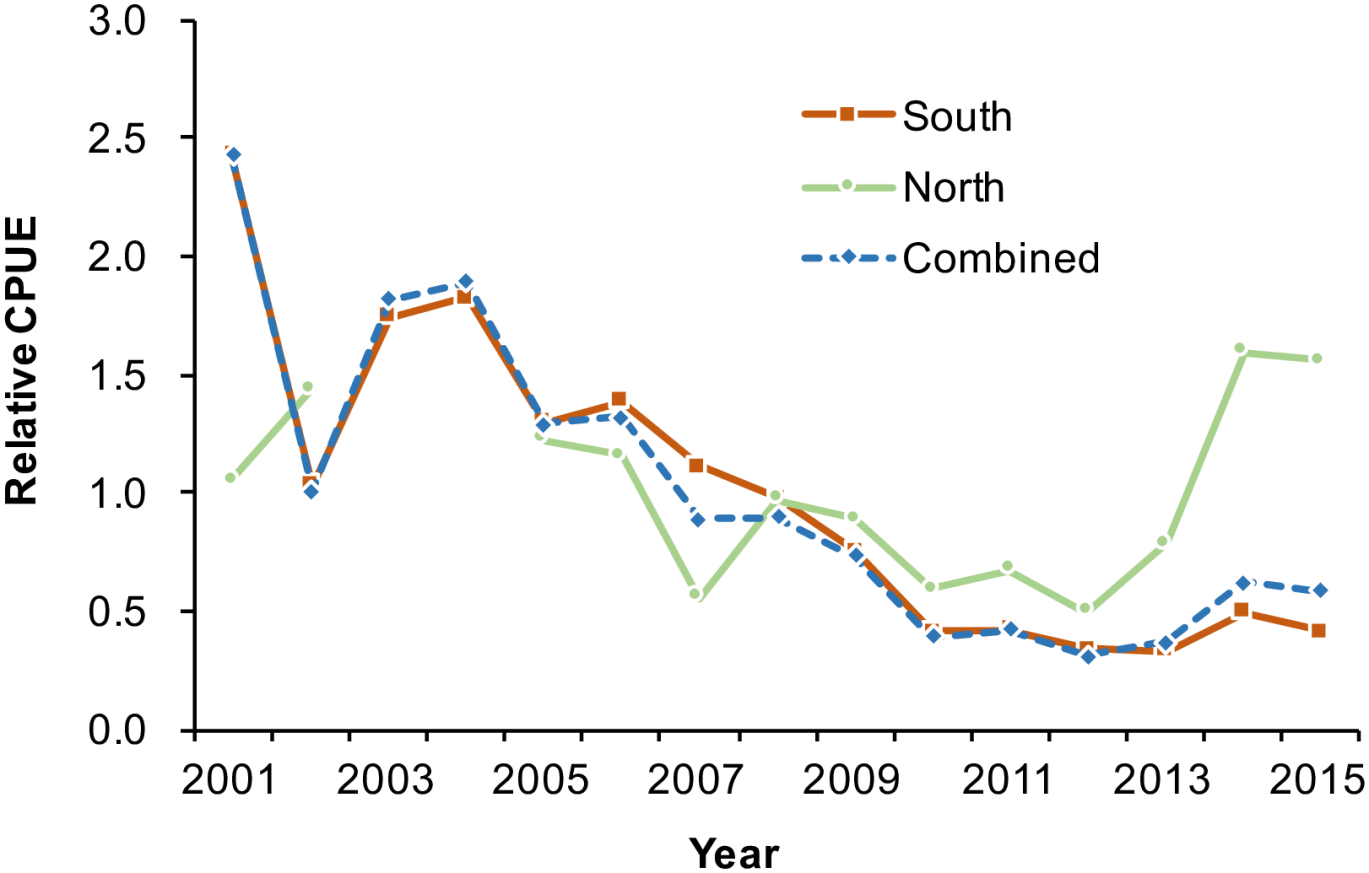
Relative standardized CPUE trends. The trends were determined from GLM runs with a delta-lognormal assumption for the area-separated model (standardizations conducted separately on southern and northern fishing grounds) and for the area-combined model (area is a variable in the standardization model). Data are provided in S6 File.

The aforementioned procedures addressed the incomplete data situation and made the CPUE of the whole series available for deriving a standardized index. By using the backward method for selecting primary explanatory variables and the forward method for selecting first-order interaction variables, the optimal variable combinations for the area-separated and area-combined standardizations were decided by the smallest AIC and BIC values (Table 4), the first-model evaluation method. Typically, all most effective models include the key variable year, month, and year × month interaction (random variable). The vessel size variable did not have the expected significant effect. The diagnostic residual plots for the standardizations separated by the fishing ground (Fig 11) indicated the appropriateness of the two-stage delta-lognormal model for evaluating the factors influencing the PBF catch rate. The resultant relative CPUE series are shown in Fig 12 (S6 File).

**Table 4:** Optimal variable combinations of the delta-lognormal mixed models that meet the model selection criteria (smallest AIC and BIC values).

Model type	Final model formulation	n	AIC	BIC
***Southern fishing ground***				
Zero-proportion model	Year + Month + Year × Month	148	419.1	422.7
Positive-catch model	Year + Month + Year × Month	6396	18696.0	18696.6
***Northern fishing ground***				
Zero-proportion model	Year + Month + Vessel size + Year × Month	106	298.3	301.6
Positive-catch model	Year + Month + Year × Month	1479	3779.9	3782.7
***Combined southern and northern fishing grounds***				
Zero-proportion model	Year + Month + Area + Year × Month	254	846.4	850.1
Positive-catch model	Year + Month + Area + Year × Month	7875	22723.4	22727.0

The GLM-based method with lognormal assumption for CPUE standardization was used because this method is commonly used for highly migratory species (e.g., tunas and billfishes) [31,40,46] and was applied in a previous study on PBF CPUE standardization in 2014 [62]. The residual plots (Fig 11) show that the distribution for the positive catches conforms well to the assumed lognormal distribution, indicating that the delta-GLM approach fits the data well. However, many other models can be used to fit the data. For example, because a single PBF catch is common, and the CPUE unit represents the number of fish in a trip, the zero-inflated negative binomial or Poisson model (non-negative integer-based distributions) would seem to be a good choice. The negative binomial model would be preferable because its mean–variance relationship generalizes that of the Poisson model.

Random effects are typically introduced into CPUE standardization models to address interactions between the year and other categorical variables that result in random changes in the population [31]. In this study, interactions of year × month and year × vessel size were both considered random variables, but only the models with year × month as random variables could converge. This practice has been used in CPUE standardization for Atlantic bluefin tuna, wherein year × month was considered a random variable [44], probably because the PBF distribution has temporal changes in the fishing season. However, the appropriateness of treating interactions between the year and other categorical variables as random effects is arguable in many situations, such as when the interaction cannot be completely explained as a random effect (e.g., it shows a significant trend) [63]. Campbell [63] typically concluded that interactions between the year and other variables should be fitted as fixed effects and reported a method for extracting the year effect from the interactions for the analyzed cases [63]. In this study, considering year × month as a fixed variable either could not yield convergent results or the obtained AIC and BIC values were large. Therefore, although arguable, this study still considered year × month as a random variable to show the effects of data reconstruction on abundance index estimation.

Two important factors should be considered in the model variables but cannot be included in the standardization model because of insufficient information are the target and habitat effects. PBF is a seasonal target species for the Taiwanese longline fishery. However, in seasons when the PBF catch is low, registered PBF vessels might shift to fish for other tuna species to increase profits. Therefore, target information is useful in the standardization process. However, because no logbook data were available for the period before 2010, a complete series of target information could not be obtained. The fish availability was low in all PBF vessels, and 99% of the trips caught 0–2 fish per day; therefore, we expect the effect to be insubstantial. Lynch et al. [40] concluded that the most accurate approach for estimating abundance indices for highly migratory species is the delta-lognormal GLM with a habitat factor. However, the habitat information is unavailable for this stock. As described earlier, PBF migrate to Taiwan waters for spawning, and the habitat depth was considered similar in all surface waters; therefore, the habitat factor might be ignorable.

#### (2) Second-stage model evaluation

When statistical models are fitted, adding parameters may increase the likelihood of overfitting; both AIC and BIC aim to address this problem by introducing a penalty term for the number of parameters to deal with the trade-off between the goodness-of-fit and complexity of the model [64]. However, the resulting AIC and BIC (Table 4) cannot be directly combined for the comparison of the area-separated model with the area-combined analysis in model selection. This is because separately applying the models to each area implies a different variance parameter for each area, and it remains unclear whether the variance parameter should be counted in the AIC and BIC. Therefore, we only assumed that considering the large difference in the AIC and BIC (e.g., the combined AIC for area-separated standardization is 376.5 smaller than that for the area-combined one), area-separated standardization was likely to have a statistically significant improvement.

An alternative approach for comparing the model fit is to calculate the *R*^*2*^ value directly from the observed and model-predicted CPUE. In this study, considering the difficulty in using the AIC and BIC for optimal model selection, the overall *R*^*2*^ value could be a more straightforward approach for model performance comparison. To avoid the overfitting effect, the second model evaluation method used cross-validation and bootstrap procedures to determine the overall *R*^*2*^ value. The results revealed that area-separated standardization yielded higher overall *R*^*2*^ (mean = 0.212, 95% confidence interval [CI] = 0.187–0.238) than did the area-combined one (mean = 0.175, 95% CI = 0.150–0.198), suggesting that area-separated standardization has a more favorable fitting performance than the area-combined one.

Furthermore, the CPUE series of the northern fishing ground revealed a different relative abundance trend than did that of the southern fishing ground, although the combined CPUE series seems to be intermediate to that of both series, particularly in the most recent 3 years (Fig 12). Most historical PBF fishing occurred in the southern fishing ground, with 90%–100% of PBF catches recorded as being from this area before 2009. The catch mainly included large fish (>210 kg, Fig 7). Thereafter, the availability of large fish declined, and gradually, more effort was deployed in the northern fishing ground, where comparatively more medium-sized fish are distributed (Fig 5) [28]. Therefore, fishing in the northern fishing ground was inconsistent over the years. Moreover, the clear difference in the size composition data by the fishing ground (Fig 5) indicated that they have different selectivities, and they caught different cohorts that migrated to the respective fishing grounds. With these considerations, area-separated standardization might be a more efficient approach.

#### (3) Third-stage model evaluation

**Fig 13:**
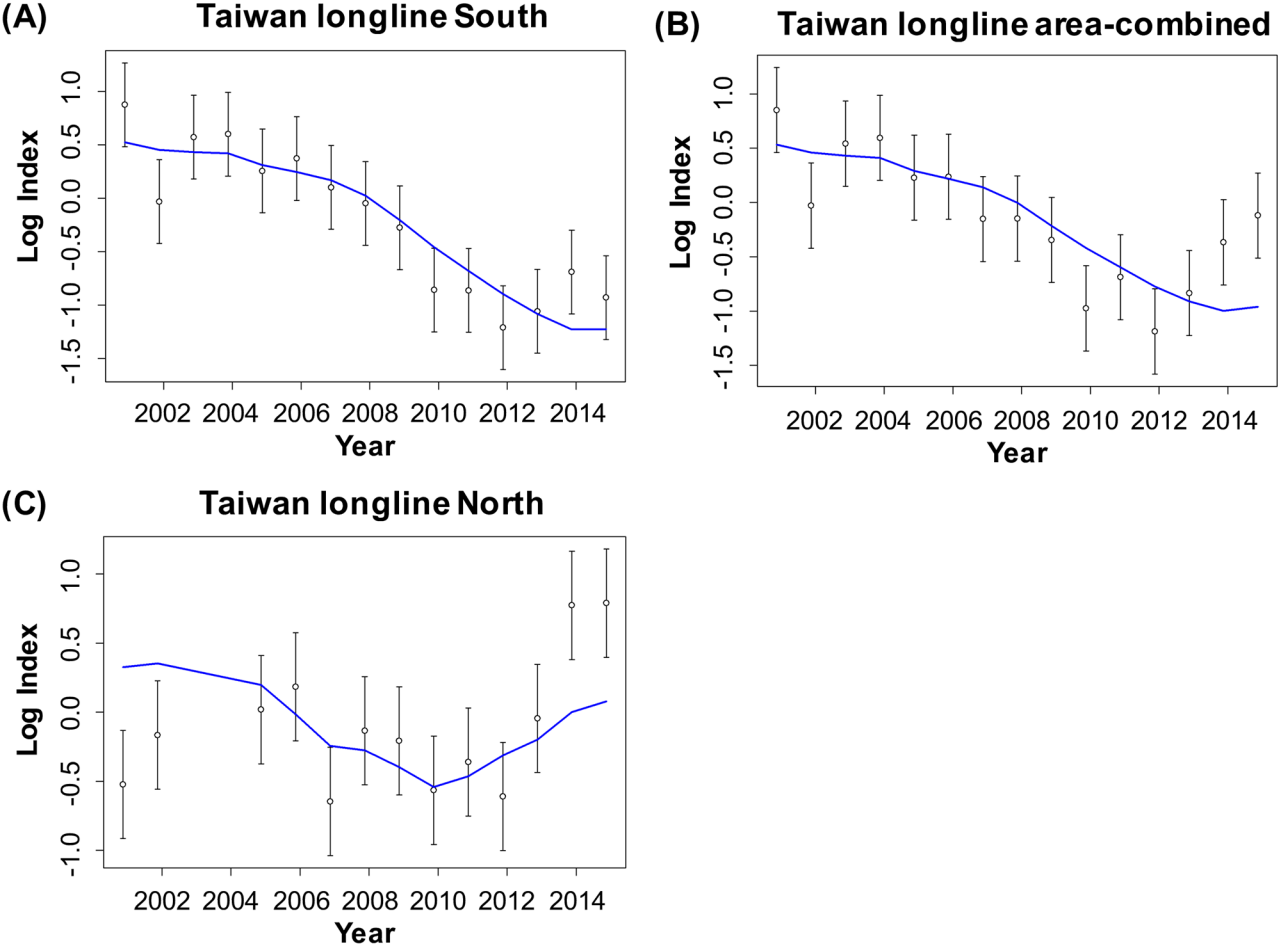
Stock Synthesis III (SS3) fit to the standardized PBF CPUE series of the Taiwanese longline fishery. The plots show model fit to the CPUE of the (A) southern fishing ground by scenario 1, (B) area-combined by scenario 2, and (C) northern fishing ground by scenario 3. Open circles with vertical error bars indicate standardized CPUE in Fig 12, and blue lines indicate the expected fit.

The third model evaluation method is system-based testing through application of the three standardized CPUE series (Table 4) to the PBF stock assessment model. The stock assessment model generally fits well to the CPUE series of the southern fishing ground (Fig 13A) when those data are included in the likelihood function of the assessment model (Table 5, scenarios 1, 3, and 5). However, the assessment model could not accurately replicate the trends of the CPUE series of the northern fishing ground and the area-combined series (Fig 13B and 13C; scenarios 2 and 3). Scenarios 2 and 3 fit even worse to the Japanese longline CPUE and the size composition data compared to scenario 1. Moreover, the CPUE series of the northern fishing ground revealed a poor fit when not included in the assessment model objective function (RMSE > 0.7), indicating that rest of the data in the stock assessment model (e.g., size composition and CPUEs of other fisheries) did not support the information derived from the CPUE series of the northern fishing ground (scenarios 1, 2, 4, and 5). The model that included only the Taiwanese southern or Japanese longline CPUE (scenarios 4 and 5) showed the lowest RMSE among the scenarios for each CPUE, whereas scenario 5 showed the lowest negative log likelihood for the size composition component.

**Table 5:** RMSEs for the SS3 model fit to the five tested scenarios.

		Abundance index RMSE				Total likelihood for size component
Scenario		TWN_South	TWN_Combined	TWN_North	JPN	
1	TWN_South and JPN CPUEs	0.273	0.438	0.867	0.265	1394
2	TWN_Combined and JPN CPUEs	0.255	0.376	0.778	0.298	1396
3	TWN_South, _North and JPN CPUEs	0.262	0.401	0.439	0.296	1415
4	Only JPN CPUE	0.340	0.521	0.944	0.237	1394
5	Only TWN_South CPUE	0.218	0.318	0.729	0.491	1385

TWN and JPN indicate Taiwanese and Japanese longline fisheries. Gray cells are the RMSEs estimated using the SS3 model that did not include the corresponding CPUE in its likelihood function.

This system-based testing method examines the diverse available information beyond catch and effort data and is based on the consistency of standardized CPUE estimates with auxiliary information. The results clearly support the CPUE series of the southern fishing ground according to the fit of the assessment model to that data as well as its consistency with the other data in the assessment model, such as the Japanese longline other fisheries’ CPUEs and size composition data. In particular, the comparison of scenarios 4 and 5 indicated that the size composition data support the CPUE series of the Taiwanese southern fishing ground over the Japanese longline CPUE. Contrastingly, the assessment model does not support the information derived by the CPUE series of the northern fishing ground. This fishing ground has been developed in recent years. Therefore, some factors might still be related to PBF catchability and should be explored and considered in the standardization procedures, such as the finer spatial and temporal effect on PBF availability from the interaction of PBF migration and fishing gear position.

The RMSE for the fit to the Taiwanese CPUE estimated in this study was 0.27, whereas a previous assessment conducted in 2014 revealed a higher RMSE (0.41), which was estimated and standardized using different data and methods (e.g., area-combined standardization) [27]. Notably, less data conflict is present in the assessment model, which was not the case in previous assessments [30]. These results indicated that the relative abundance information derived from the standardized CPUE of the southern fishing ground estimated in this study is more consistent with the assumed PBF population dynamics and other fishery data than is the standardized CPUE estimated by the previous study; therefore, this series was selected by the PBF Working Group of the ISC to be included in the latest base case assessment model of PBF [13].

Based on these results and qualitative consistency in the fishing activity and the fish size caught in each fishing ground within the PBF distribution range, the system-based testing demonstrated that data reconstruction has improved the abundance index estimation and, consequently, has addressed the concerns of data inconsistency indicated in the previous PBF stock assessment. The PBF stock assessment model is an integrated stock assessment model which has become the dominant method for assessing tuna stocks. The model represents scientific understanding of the dynamics of the population in equations that define how the population and its structure changes over time and uses all available information about the population [65]. Therefore, the improvement in consistency of the reconstructed index with other information is also an improvement to the stock assessment of the stock.

## Supporting information

S1 File PBF catch data for 1952–2015 by country and fisheries, and catch data for the Taiwanese longline fishery during 1997–2015. Data for 2015 are preliminary.
(XLSX)

S2 File Length frequency data for PBF caught by the Taiwanese longline fishery. (XLS)

S3 File Detailed information on the model structure, fishery data and assumptions, and biological assumptions for the PBF stock assessment model by using Stock Synthesis version 3.24f. (XLSX)

S4 File Average PBF weight data standardized by the category of Taiwanese PBF catches for 2004–2015. (XLSX)

S5 File At-sea and fishing day data for estimating the linear relationships presented in Table 3. (XLSX)

S6 File CPUE data standardized by fishing grounds for the Taiwanese longline fishery. (XLSX)
